# Antimicrobial Spectrum of Activity and Mechanism of Action of Linear Alpha-Helical Peptides Inspired by Shrimp Anti-Lipopolysaccharide Factors

**DOI:** 10.3390/biom13010150

**Published:** 2023-01-11

**Authors:** Gabriel Machado Matos, Beatriz Garcia-Teodoro, Camila Pimentel Martins, Paulina Schmitt, Fanny Guzmán, Ana Claudia Oliveira de Freitas, Patricia Hermes Stoco, Fabienne Antunes Ferreira, Marciel João Stadnik, Diogo Robl, Luciane Maria Perazzolo, Rafael Diego Rosa

**Affiliations:** 1 Laboratory of Immunology Applied to Aquaculture, Department of Cell Biology, Embryology and Genetics, Federal University of Santa Catarina, Florianópolis 88040-900, Brazil; 2 Laboratorio de Genética e Inmunología Molecular, Instituto de Biología, Facultad de Ciencias, Pontificia Universidad Católica de Valparaíso, Valparaíso 2373223, Chile; 3 Núcleo Biotecnología Curauma, Pontificia Universidad Católica de Valparaíso, Valparaíso 2373223, Chile; 4 Laboratory of Protozoology, Department of Microbiology, Parasitology and Immunology, Federal University of Santa Catarina, Florianópolis 88040-900, Brazil; 5 Laboratory of Molecular Genetics of Bacteria, Department of Microbiology, Parasitology and Immunology, Federal University of Santa Catarina, Florianópolis 88040-900, Brazil; 6 Laboratory of Plant Pathology, Department of Plant Sciences, Federal University of Santa Catarina, Florianópolis 88034-001, Brazil; 7 Laboratory of Microorganisms and Biotechnological Processes, Department of Microbiology, Parasitology and Immunology, Federal University of Santa Catarina, Florianópolis 88040-900, Brazil

**Keywords:** crustacean, antimicrobial peptide (AMP), host defense peptide (HDP), antibacterial, antifungal, antiparasitic activity, methicillin-resistant *Staphylococcus aureus* (MRSA), membrane-disrupting, synergy, cytotoxicity

## Abstract

Shrimp antilipopolysaccharide factors (ALFs) form a multifunctional and diverse family of antimicrobial host defense peptides (AMPs) composed of seven members (groups A to G), which differ in terms of their primary structure and biochemical properties. They are amphipathic peptides with two conserved cysteine residues stabilizing a central β-hairpin that is understood to be the core region for their biological activities. In this study, we synthetized three linear (cysteine-free) peptides based on the amino acid sequence of the central β-hairpin of the newly identified shrimp (*Litopenaeus vannamei*) ALFs from groups E to G. Unlike whole mature ALFs, the ALF-derived peptides exhibited an α-helix secondary structure. In vitro assays revealed that the synthetic peptides display a broad spectrum of activity against both Gram-positive and Gram-negative bacteria and fungi but not against the protozoan parasites *Trypanosoma cruzi* and *Leishmania* (*L*.) *infantum*. Remarkably, they displayed synergistic effects and showed the ability to permeabilize bacterial membranes, a mechanism of action of classical AMPs. Having shown low cytotoxicity to THP-1 human cells and being active against clinical multiresistant bacterial isolates, these nature-inspired peptides represent an interesting class of bioactive molecules with biotechnological potential for the development of novel therapeutics in medical sciences.

## 1. Introduction

The selection of microorganisms with resistance against multiple conventionally used antibiotics represents one of the leading health problems worldwide [1]. The study of antimicrobial host defense peptides (AMPs) has recently expanded and emerged as an alternative for developing new antimicrobials and treating multidrug-resistant bacteria [2,3]. AMPs are natural defense molecules found in virtually all kingdoms of life, from single-celled microbes to mammals. Classically, they are described as peptides of less than 10 kDa, usually cationic, that selectively target the negatively charged membranes of microorganisms [4]. Preclinical trials have demonstrated the potential of AMPs for control and prevention of several clinical conditions, such as the treatment of bacterial infections in burns [5], restoration of lung function after pneumonia [6], and inhibition of biofilms [7]. Due to the success of these tests, some AMPs have been approved and tested in clinical trials, thus endorsing their potential as a novel class of antibiotics (for reviews, see [2,8]).

Crustaceans are one of the largest and most diverse animal groups on Earth. They are exposed to abundant and changing microbial communities in their natural environments, which include both commensals and opportunistic pathogens. To cope with this microbial diversity, crustaceans display an entire arsenal of immune defense mechanisms and produce efficient antimicrobial compounds, including a wide range of AMPs [9]. To date, 12 gene-encoded AMP families and other antimicrobial-related molecules (e.g., lysozymes, multifunctional proteins, and nonribosomally synthesized AMPs encrypted in large proteins) are recognized in crustaceans [9]. Most of these AMPs comprise multigene families and display a diverse spectrum of antimicrobial activity, highlighting the great diversity of AMPs in crustaceans (for reviews, see [9,10]).

Anti-lipopolysaccharide factors (ALFs or anti-LPS factors) represent a highly diverse multigene family found in most crustaceans and exhibit the broadest spectrum of antimicrobial activity compared to other AMPs found in this taxa [9]. In penaeid shrimp, the ALF family is composed of seven members (designated as ‘groups’) displaying distinct biochemical features: groups A, D, E, and G are anionic, whereas groups B, C, and F are cationic [9,11]. This diversity is encoded by at least seven genes that arose from successive duplications and subsequent mutations (nucleotide substitutions and insertion/deletion events) before decapod crustacean speciation occurred [11]. This indicates that shrimp ALFs are paralogous genes that evolved before the speciation of the suborder Dendrobranchiata (penaeid shrimp) and that strong evolutionary pressures have driven the functional diversification of ALF genes in penaeid shrimp [11]. It has been shown that the amino acid sequence variability in each ALF group contributes to the functional divergence described for this AMP family [11,12]. While some members exhibited a broad range of antimicrobial activity (groups B and G), some others displayed limited (groups A, C, and E) or very weak action (groups D and F) [11,12,13].

ALFs are amphipathic AMPs with a highly hydrophobic N-terminal region and two conserved cysteine residues. The three-dimensional structure of ALFs consists of three α-helices packed against a four-stranded β-sheet, while the two cysteines stabilize a central β-hairpin [14]. This central β-hairpin is considered the core region for ALF biological activity. Indeed, the antimicrobial mechanism of action of ALFs is intimately associated with their ability to bind to microbial moieties, such as lipoteichoic acid (LTA) from Gram-positive bacteria, LPS from Gram-negative bacteria, and β-glucans from fungi [15]. Interestingly, synthetic peptides based on the central β-hairpin of shrimp ALFs were found to display antimicrobial activity similar to the whole mature molecule [11,12,16,17], which is what facilitates the production and application of these AMPs. In addition, substitutions of uncharged with cationic residues led to an increase in the antimicrobial activity of β-hairpin-derived peptides, confirming that modifications in the primary structure can result in peptides with enhanced antimicrobial properties [17,18].

ALFs from groups A to D were the first members described in penaeid shrimp and have been widely studied in recent decades [12,13,16,19,20]. Therefore, we decided to explore the potential of the three most recently described and poorly known shrimp ALFs from groups E to G for drug discovery [11]. We show here that synthetic linear α-helical peptides based on the central β-hairpin of *Litopenaeus vannamei* ALFs from groups E to G exhibit a broad spectrum of antibacterial and antifungal activities, including multiresistant clinical isolates. These synthetic peptides were able to act synergistically to inhibit the growth of both Gram-positive and Gram-negative bacteria and yeast. However, they were not active against protozoan parasites. Finally, shrimp ALF-derived peptides could permeabilize bacterial membranes and showed low cytotoxicity to human cells. These results emphasize the biotechnological potential of this AMP family for the development of novel antibiotics.

## 2. Materials and Methods

### 2.1. Bacterial and Fungal Strains

In this study, we used different bacterial and fungal strains of agricultural, biotechnological, and veterinary/human health interest. *Corynebacterium stationis* CIP 101282, *Escherichia coli* SBS 363, *Microbacterium maritypicum* CIP 105733, *Micrococcus luteus* CIP 5345, and *Vibrio nigripulchritudo* CIP 103195 were obtained from the Collection of the Pasteur Institute (CIP, Paris, France). *Aspergillus brasiliensis* ATCC 16404, *Bacillus subtilis* ATCC 6633, *Candida krusei* ATCC 6258, *Candida parapsilosis* ATCC 22019, *Enterococcus faecalis* ATCC 29212, *Pseudomonas aeruginosa* ATCC 9027, *Staphylococcus aureus* ATCC 25932 and ATCC 29737, *Trichoderma virens* ATCC 9645, *Vibrio alginolyticus* ATCC 17749, *Vibrio anguillarum* ATCC 19264, and *Vibrio harveyi* ATCC 14126 were obtained from the American Type Culture Collection (ATCC, Manassas, VA, USA). *Fusarium oxysporum* MUCL 909 and *S. aureus* SG511 were obtained from the University of Montpellier (a generous gift from Dr. Delphine Destoumieux-Garzón, Montpellier, France), while *Candida albicans* 12A (MDM8) was obtained from the Butantan Institute (a generous gift from Dr. Pedro Ismael da Silva Jr., São Paulo, Brazil). *Vibrio parahaemolyticus* IOC 18950 was obtained from the Oswaldo Cruz Institute (Rio de Janeiro, Brazil). The filamentous fungi *Aspergillus niger* LAMPB-UFSC DR02 and *Rhizopus* sp. LAMPB-UFSC were obtained from the fungi culture collection of the Laboratory of Microorganisms and Biotechnological Processes (Federal University of Santa Catarina, Florianópolis, Brazil), while *Colletotrichum chrysophilum* MANE 147 and *Colletotrichum higginsianum* MANE 166 were obtained from the Micoteca Anne Löre Schroeder (Federal University of Santa Catarina, Florianópolis, Brazil). *Candida glabrata* CCT 0728 was obtained from the Collection of Tropical Cultures (Fundação André Tosello, São Paulo, Brazil), while *Candida tropicalis* LMC-UFSC was obtained from the Laboratory of Clinical Mycology (a generous gift from Dr. Jairo Ivo dos Santos, Federal University of Santa Catarina, Florianópolis, Brazil). *Saccharomyces cerevisiae* CAT1 was kindly given by Dr. Boris Stambuk (Federal University Santa Catarina, Florianópolis, Brazil).

The vibrios *V. fluvialis* EMBRAPA-SE and *V. parahaemolyticus* EMBRAPA-SE (a generous gift from Dr. Alitiene Pereira, Empresa Brasileira de Pesquisa Agropecuária, Aracaju, Brazil), the yeast *Rhodotorula* sp. LIAA-UFSC (Laboratory of Immunology Applied to Aquaculture, Federal University of Santa Catarina, Florianópolis, Brazil), and the filamentous fungus *Penicillium* sp. LIAA-UFSC were isolated from the Pacific white shrimp *L. vannamei*. The methicillin-resistant *S. aureus* (MRSA) strains were isolated from hospitalized patients and kindly given by Dr. Thais Sincero (Federal University Santa Catarina, Florianópolis, Brazil). MRSA isolation was approved by the Human Research Ethics Committee from UFSC. Media and culture conditions are listed in Table S1 as supplementary material.

### 2.2. Chemical Synthesis of ALF-Derived Peptides

Three short, linear, cysteine-free peptides were chemically synthetized based on the amino acid sequence of the central β-hairpin of shrimp (*L. vannamei*) ALFs from groups E to G [11]. The synthetic 20 residue peptides were obtained in a Liberty Blue automated microwave peptide synthesizer (CEM Corp, Matthews, NC, USA) using Fmoc-protected amino acids (Iris Biotech GmbH (Marktredwitz, Germany)) and Rink Amide AM resin (loading: 0.6 meq/g). Fmoc deprotection was carried out with 20% *v*/*v* piperidine in DMF (N, N-dimethylformamide), couplings were performed with DIC/OxymaPure activation (1/1 eq), and additional couplings with TBTU/DIEA/OxymaPure activation (1/2/1 eq). Peptides were cleaved with TFA/TIS/DOT/H_2_0 (92.5/2.5/2.5/2.5) (trifluoroacetic acid/triisopropylsilane/2,2-(ethylenedioxy)-diethanethiol/ultrapure water) and purified with RP-HPLC (JASCO Corp., Tokyo, Japan) on an XBridge™ BEH C18 column (100 × 4.6 mm, 3.5 μm) (Waters Corp., Milford, MA, USA) with a 0–70% acetonitrile–water mixture gradient over 30 min at a flow rate of 1 mL/min. Peptides were further lyophilized and analyzed with matrix-assisted laser desorption ionization—time of flight (MALDI-TOF) mass spectrometry in an LCMS-2020 ESI-MS (Shimadzu Corp., Kyoto, Japan) to confirm their molecular masses.

Crude peptide (50 mg) was first reduced with 10% β-mercaptoethanol (95 °C for 5 min) then dissolved in 50% (*v*/*v*) AcOH/H_2_O and later diluted in 32 mL of oxidation buffer (2 mM guanidinium chloride, 10% isopropyl alcohol, and 10% dimethyl sulfoxide). The pH was adjusted to 5.8 with ammonium hydroxide. The peptide solution was subjected to air oxidation at room temperature for 18 h. The peptide solution was then acidified to pH 2.5 and purified using a SPE C18 (Waters Corp., Milford, MA, USA). The peptides were eluted with 5%, 20%, 40%, 60%, and 80% acetonitrile in 0.05% TFA ultrapure water at a flow rate of 1 mL/min. The fractions were collected, and the acetonitrile was evaporated on a Savant SPD 1010 SpeedVac Concentrator (Thermo Scientific, Asheville, NC, USA). The fractions were analyzed with MALDI-TOF mass spectrometry.

### 2.3. Circular Dichroism (CD) Measurement

Circular dichroism (CD) spectroscopy was carried out on a JASCO J-815 CD Spectrometer coupled to a Peltier JASCO CDF-426S/15 system for temperature control (JASCO Corp., Tokyo, Japan) in the far ultraviolet range (λ = 190–250 nm), using quartz cuvettes with a 0.1 cm path length and 1 nm bandwidth at 0.1 nm resolution. Each spectrum was recorded as an average of four scan repetitions in continuous scanning mode with 50 nm/min scanning speed and a response time of 1 s. The solvent contribution blank was subtracted from each sample spectrum. Molar ellipticity was calculated for each synthetic peptide. CD spectra of the peptides were recorded in ultrapure water and trifluoroethanol (TFE, 30% *v*/*v* in ultrapure water). The spectra were recorded at 20, 30, and 37 °C.

### 2.4. Antibacterial Assays

The antibacterial activity of the synthetic ALF-derived peptides was assayed against reference, clinical, and environmental strains of Gram-positive and Gram-negative bacteria (phyla Actinobacteria, Firmicutes, and Proteobacteria). Minimum inhibitory concentrations (MICs) were determined in duplicate with the liquid growth inhibition assay in 96-well microtiter plates, as previously described [21]. In brief, 10 μL of peptides (final concentration, range from 40 to 1.25 μM) was incubated with 90 μL of bacterial suspension brought to the exponential growth phase and adjusted to A_600nm_ = 0.001 in its respective media and culture conditions under shaking (Table S1). In negative controls, peptides were substituted with sterile ultrapure water. Growth was monitored spectrophotometrically (λ = 600 nm) on a Tecan Infinite M200 spectrophotometer (Tecan, Männedorf, Switzerland) at 24 h. MIC values are expressed as the lowest concentration tested (in μM) that caused 100% growth inhibition. The minimum bactericidal concentration (MBC) was determined by plating 100 μL of overnight cultures onto nutrient agar plates from each of the wells from the MIC test that showed no turbidity. The plates were incubated for 48 h. MBC values were expressed as the lowest concentration tested (in μM) for which no bacterial growth was observed on the plates.

### 2.5. Antifungal Assays

The antifungal activity of the synthetic ALF-derived peptides was assayed against reference and environmental strains of filamentous fungi and yeasts (phyla Ascomycota, Basidiomycota, and Mucoromycota). The MIC for filamentous fungi was determined in duplicate with the liquid growth inhibition assay using spores, as previously described [12]. In brief, pure cultures were grown in potato dextrose agar (Table S1) at 28 °C until abundant sporulation. Spores were harvested with 0.1% Tween-20 (*v*/*v* in sterile ultrapure water), filtered through lint, and centrifuged (3000× *g* for 15 min). Spore pellets were washed twice with 0.1% Tween-20, and their concentration was adjusted in a Neubauer chamber. For the tests, 90 μL of fungal spores (final concentration, 10^4^ spores/mL) suspended in potato dextrose broth at half-strength (½ PDB, Table S1) were added to 10 μL of peptides (final concentration, range from 40 to 1.25 μM) in 96-well microtiter plates. In negative controls, peptides were substituted with sterile ultrapure water. Spore germination was observed under an inverted microscope after 48 h of incubation with shaking in a humidity chamber at 28 °C in the dark. The minimum fungicidal concentration (MFC) was determined by plating 100 μL of fungal cultures onto PDA plates from each of the wells from the MIC test that showed no spore germination. The plates were incubated for 48 h at 28 °C. MFC values were expressed as the lowest concentration tested (in μM) for which no growth was observed on PDA plates.

The MIC values for the antiyeast assays were determined as described for the antibacterial tests. In brief, 10 μL of peptides (final concentration, range from 40 to 1.25 μM) were incubated with 90 μL of yeast suspension brought to the exponential growth phase and adjusted to A_600nm_ = 0.001 in Sabouraud medium (Table S1) at 28 °C under shaking conditions. In controls, peptides were substituted by sterile ultrapure water. Growth was monitored spectrophotometrically (λ = 600 nm) on a Tecan Infinite M200 spectrophotometer (Tecan, Männedorf, Switzerland) at 48 h. MIC values are expressed as the lowest concentration tested (in μM) that caused 100% yeast growth inhibition. The MFC was determined by plating 100 μL of yeast cultures onto Sabouraud agar plates from each of the wells from the MIC test that showed no growth. Cultures were incubated for 48 h at 28 °C. MFC values were expressed as the lowest concentration tested (in μM) for which no growth was observed on the plates.

### 2.6. Determination of Fractional Inhibitory Concentrations (FICs)

The synergic effect of the synthetic ALF-derived peptides in inhibiting the growth of the bacterial strains *M. maritypicum* CIP 105733 and *E. coli* SBS 363 and the shrimp midgut-associated yeast *Rhodotorula* sp. LIAA-UFSC was evaluated using the titration test [22] with some modifications. To assess the reduction in the inhibitory concentration of each peptide, serial dilutions of one peptide (peptide ”A”) were combined with half the MIC of the other peptide (peptide ”B”) and vice versa. The results obtained were expressed using the fractional inhibitory concentration (FIC) index. FIC values were calculated using the formula: FIC = [A]/MIC_A_ + [B]/MIC_B_, where MIC_A_ and MIC_B_ are the MICs of peptide ”A” and peptide ”B” tested individually, and [A] and [B] are the MICs of the peptides tested together. FIC values were interpreted as follows: FIC ≤ 0.5, strong synergic effect; FIC = 0.5–1, synergic effect; FIC ≥ 1, additive effect; FIC = 2, no synergic effect; FIC ≥ 2, antagonist effect. Microbial growth and incubation were performed as described in Section 2.4 and Section 2.5.

### 2.7. Assays for Bacterial Membrane Permeability

The effect of the synthetic ALF-derived peptides on the integrity of bacterial membranes was assessed with the Sytox Green uptake assay. Exponential phase cultures of *E*. *coli* SBS 363 were diluted at A_600nm_ = 0.2 in 10 mM phosphate-buffered saline (PBS) supplemented with 138 mM NaCl and 2.7 mM KCl (pH 7.4). Then, 45 μL of bacterial solution containing 1 μM Sytox Green (Invitrogen, Carlsbad, CA, USA) were dispensed into 0.1 mL MicroAmp Fast 96-well reaction plates (Thermo Scientific, Asheville, NC, USA) containing 5 μL of each peptide in triplicate (final concentration, 5 μM). In positive controls, peptides were substituted with 1.25 μM of an amidated analog of magainin, a recognized pore-forming AMP from the African clawed frog *Xenopus laevis* (amino acid sequence = GIGKFLKKAKKFGKAFVKMKK-NH2, molecular weight = 2495.97 Da, p*I* = 10.9 [23]). In negative controls, peptides were substituted with sterile ultrapure water. Sytox Green uptake was measured every 30 s over 1 h (λ excitation = 480 nm; λ emission = 550 nm) at 37 °C using a StepOnePlus Real-time PCR System (Thermo Scientific, Asheville, NC, USA).

### 2.8. Antiparasitic Activity

Cultures of *Trypanosoma cruzi* (Tulahuen strain) epimastigotes and *Leishmania* (*L*.) *infantum* (MHOM/BR/74/PP75 strain) promastigotes were grown in liver infusion tryptose (LIT) and M199 supplemented with 10% fetal bovine serum (FBS), with the latter also supplemented with 5% human urine, at 26.5 °C with 5% CO_2_ in an incubator. For the tests, 90 μL of parasite suspension (5.4 × 10^5^ parasites/well) was added to each well of 96-well microtiter plates, followed by 10 μL of peptides (final concentration, range from 20 to 1.25 μM). In negative controls, peptides were substituted with sterile ultrapure water, whereas 20 μM benznidazole (Sigma-Aldrich, St. Louis, MO, USA) and 2 μM amphotericin B (Bristol-Myers, Squibb, Woerden, Netherlands) were used as positive controls for trypanocidal and leishmanicidal activities, respectively. A sample control was included for each peptide dilution containing only the peptide and the respective parasite medium. The 96-well microtiter plates were incubated for 72 h at 26.5 °C. The antiparasitic activity was determined in triplicate with a quantitative colorimetric assay using the oxidation–reduction (blue-pink) indicator resazurin as an indicator for metabolic function. *T. cruzi* and *L*. (*L*.) *infantum* parasites were incubated for 5 h and 1 h 30 min, respectively, with 3 mM resazurin solution at 26.5 °C in the dark. Parasite viability was monitored using fluorescence quantification (λ excitation = 560 nm; λ emission = 590 nm) on a Tecan Infinite M200 spectrophotometer (Tecan, Männedorf, Switzerland). Relative viability was calculated for each sample by averaging fluorescence readings across the triplicates, subtracting the average for the sample control, and then dividing by the value obtained for the negative control.

### 2.9. Cytotoxicity Assays

The human leukemia monocytic cell line THP-1 (ATCC TIB202), obtained from the cryobank of the Laboratory of Protozoology at the Federal University of Santa Catarina (Florianópolis, Brazil), was cultured in RPMI-1640 medium supplemented with 10% FBS, 2 mM l-glutamine, and 1 mM sodium pyruvate and grown at 37 °C with 5% CO_2_ in an incubator. For the tests, in a 96-well microtiter plate, 100 μL of THP-1 cell suspension (2.2 × 10^5^ cells/mL) was differentiated into macrophages using 100 ng of phorbol-12-myristate-13-acetate (PMA) and incubated for 72 h at 37 °C with 5% CO_2_. Then, the medium was replaced with 90 μL of RPMI-1640 supplemented with 10% FBS, 2 mM l-glutamine, and 1 mM sodium pyruvate followed by 10 μL of peptides (final concentration, range from 80 to 1.25 μM), and the 96-well microtiter plate was incubated for 72 h at 37 °C. DMSO 50% and RPMI-1640 were used as positive and negative controls, respectively. Cytotoxicity was determined in triplicate with a quantitative colorimetric assay using the oxidation–reduction (blue-pink) indicator resazurin as an indicator for metabolic function. Fluorescence was measured after 24 h incubation with 3 mM resazurin solution using fluorescence quantification (λ excitation = 560 nm; λ emission = 590 nm) on a Tecan Infinite M200 spectrophotometer (Tecan, Männedorf, Switzerland). Cell viability was calculated by averaging fluorescence readings across the triplicates, subtracting the average for the sample control, and then dividing by the value obtained for the negative control.

## 3. Results

### 3.1. Linear ALF-Derived Peptides Showed an α-Helical Secondary Structure

**Figure 1:**
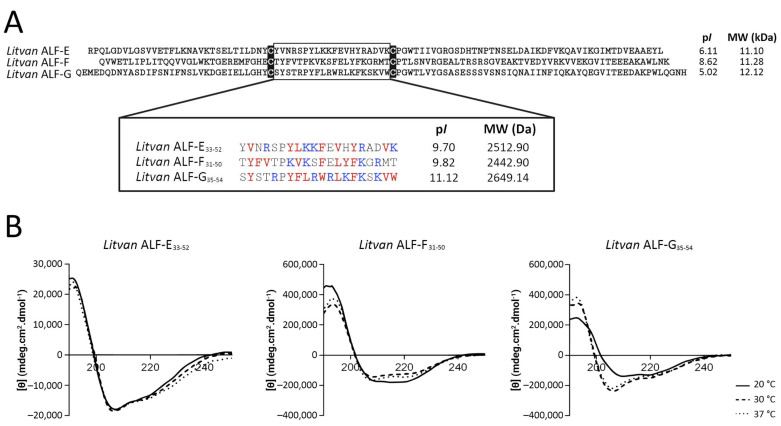
Linear, cysteine-free peptides synthetized on the basis of the central β-hairpin of shrimp (*Litopenaeus vannamei*) ALFs from groups E to G [11]. (**A**) Amino acid sequence and biochemical properties of whole mature ALFs (GenBank accession numbers, *Litvan* ALF-E: FE069658, *Litvan* ALF-F: KJ000049, *Litvan* ALF-G: GETZ01049665) and the synthetic ALF-derived peptides (*Litvan* ALF-E_33–52_, *Litvan* ALF-F_31–50_, and *Litvan* ALF-G_35–54_). The subscript numbers indicate the position of the synthetic peptides within the amino acid sequence of the whole mature ALFs. The molecular weight (MW) of the ALF-derived peptides was determined with MALDI-TOF analysis. p*I*: theoretical isoelectric point. Cationic and hydrophobic residues are highlighted in blue and red, respectively. (**B**) Circular dichroism spectra of the synthetic ALF-derived peptides.

To evaluate the potential of antilipopolysaccharide factors (ALFs) for the nature-based design of novel therapeutics, we synthesized short, linear, cysteine-free peptides (20 amino acid residues) based on the central β-hairpin (the functional domain) of three ALF members (*Litvan* ALF-E to -G) recently characterized in the penaeid shrimp *L. vannamei* [11]. This region was selected for peptide synthesis due to the presence of hydrophobic and cationic residues, resulting in high amphipathicity (Figure 1A). Remarkably, while the whole mature peptides *Litvan* ALF-E (GenBank: FE069658) and *Litvan* ALF-G (GenBank: GETZ01049665) exhibited anionic properties (p*I* 6.11 and 5.02, respectively) [11], their central β-hairpins (*Litvan* ALF-E_33–52_ and *Litvan* ALF-G_35–54_) were highly cationic (p*I* 9.70 and 11.12, respectively) (Figure 1A). An increase in the p*I* was also observed for *Litvan* ALF-F_31–50_ (Figure 1A) when compared to the mature *Litvan* ALF-F [11]. In all mature ALFs, the two conserved cysteine residues delimit a central β-hairpin structure that is considered essential for their biological activities [9].

The circular dichroism (CD) spectra of the linear, cysteine-free peptides were recorded in an aqueous solution added to TFE 30% at room temperature (20 °C) and at microorganism growth temperatures (30 and 37 °C) (Figure 1B). A standard α-helix signal with characteristic peaks at 195, 208, and 222 nm was detected for the three synthetic peptides in all temperatures (Figure 1B). These results indicate that the synthetic ALF-derived peptides have an α-helical structure similar to classical cationic antimicrobial peptides (cAMPs), which is distinct from that observed for the cysteine-stabilized β-hairpin of ALFs [14].

### 3.2. The Synthetic Litvan ALF-G_35–54_ Peptide Displays a Broad Spectrum of Antibacterial Activity

To characterize the antibacterial activity of the three synthetic ALF-derived peptides, minimal inhibitory and bactericidal concentration (MIC and MBC) assays were performed against reference (e.g., ATCC and CIP), clinical (methicillin-resistant *S. aureus*), and environmental strains of Gram-positive (n = 12) and Gram-negative (n = 9) bacteria. Among the three peptides, *Litvan* ALF-G_35–54_ displayed the broadest and the strongest antibacterial spectrum, inhibiting the growth of most tested bacteria (15/21) at low concentrations (Table 1). Notably, *Litvan* ALF-G_35–54_ was active against the clinical methicillin-resistant *S. aureus* (MRSA) isolates 16003 (resistant to cefoxitin) and 17022 (resistant to cefoxitin, ciprofloxacin, clindamycin, erythromycin, gentamicin, rifampicin, and trimethoprim-sulfamethoxazole). In addition, this synthetic peptide exhibited bactericidal activity against the Gram-positives *B. subtilis* ATCC 6633, *C. stationis* CIP 101282, *M. maritypicum* CIP 105733, *M. luteus* CIP 5345, and *S. aureus* ATCC 25932 and SG511 and against the Gram-negatives *E. coli* SBS 363, *V. harveyi* ATCC 14126, *V. nigripulchritudo* CIP 103195, and *V. parahaemolyticus* IOC 18950 (Table 1).

*Litvan* ALF-F_31–50_ displayed bacteriostatic and bactericidal activities against *C. stationis* CIP 101282, *M. maritypicum* CIP 105733, *M. luteus* CIP 5345, *E. coli* SBS 363, and *V. nigripulchritudo* CIP 103195 (Table 1), while *Litvan* ALF-E_33–52_ was only active against the marine Gram-positive *M. maritypicum* CIP 105733 (Table 1). Finally, none of the synthetic peptides were able to inhibit the growth of the Gram-positive *Enterococcus faecalis* ATCC 29212, the MRSA isolates 16006 (resistant to cefoxitin, ciprofloxacin, clindamycin, and erythromycin) and 17018 (resistant to cefoxitin, ciprofloxacin, clindamycin, erythromycin and gentamicin), the Gram-negative *V. alginolyticus* ATCC 17749, and the two environmental vibrios isolated from diseased shrimp (*V. fluvialis* EMBRAPA-SE and *V. parahaemolyticus* EMBRAPA-SE) (Table 1).

### 3.3. Antifungal Activity of Shrimp ALF-Derived Peptides Is Mainly Directed toward Yeasts

The antifungal activity of synthetic ALF-derived peptides was assayed against reference (e.g., ATCC and MANE) and environmental strains of filamentous fungi (n = 8) and yeasts (n = 7) (Table 2). Regarding the filamentous fungi, only *Litvan* ALF-G_35–54_ could completely inhibit the spore germination of *F. oxysporum* MUCL 909, *Rhizopus* sp. LAMPB-UFSC, and the shrimp midgut-associated strain *Penicillium* sp. LIAA-UFSC (Table 2). This peptide exhibited fungicidal activity at the same concentration against *F. oxysporum* MUCL 909. None of the synthetic peptides showed antifungal activity against *A. brasiliensis* ATCC 16404, *A. niger* LAMPB-UFSC DR02, *C. chrysophilum* MANE 147, *C. higginsianum* MANE 166, and *T. virens* ATCC 9645 (Table 2).

Regarding the yeasts, all peptides were active against *Candida tropicalis* LMC-UFSC, *Rhodotorula* sp. LIAA-UFSC, and the industrial fuel-ethanol fermentative *S. cerevisiae* CAT1 (Table 2). In addition, *Litvan* ALF-G_35–54_ was also active against *C. krusei* ATCC 6258 and *C. parapsilosis* ATCC 22019 (Table 2). Yeasticidal activity was observed for *Litvan* ALF-G_35–54_ on *C. krusei* ATCC 6258 and on *Rhodotorula* sp. LIAA-UFSC, for *Litvan* ALF-F_31–50_ and *Litvan* ALF-G_35–54_ on *C. tropicalis* LMC-UFSC, and for *Litvan* ALF-E_33–52_ and *Litvan* ALF-G_35–54_ on *S. cerevisiae* CAT1 (Table 2). Finally, none of the synthetic peptides inhibited the growth of *C. albicans* 12A (MDM8) and *C. glabrata* CCT 0728 (Table 2).

### 3.4. Shrimp ALF-Derived Peptides Can Act Synergically to Inhibit Microbial Growth

To gain evidence of potential synergy between the shrimp ALF-derived peptides, we measured synergistic in vitro activities against the bacterial strains *M. maritypicum* CIP 105733 and *E. coli* SBS 363 and the yeast *Rhodotorula* sp. FIC indexes were calculated for distinct combinations of synthetic peptides using the checkerboard assay. Results showed that *Litvan* ALF-F_31–50_ and *Litvan* ALF-G_35–54_ acted synergistically against bacteria (FIC = 0.5–1) but not against the yeast *Rhodotorula* sp. (FIC = 2) (Table 3). In contrast, *Litvan* ALF-E_33–52_ and *Litvan* ALF-F_31–50_ acted synergistically against the yeast but not against the bacteria (Table 3). Finally, the combination of *Litvan* ALF-E_33–52_ and *Litvan* ALF-G_35–54_ (FIC = 0.63) led to an eightfold reduction in the concentration of *Litvan* ALF-E_33–52_ required to inhibit the growth of *M. maritypicum* CIP 105733 (Table 1 and Table 3).

### 3.5. Shrimp ALF-Derived Peptides Permeabilize Bacterial Membranes

**Figure 2:**
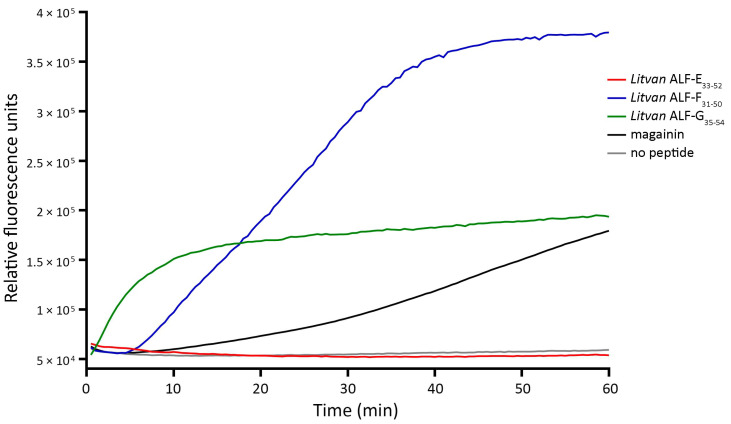
Membrane permeabilization of *Escherichia coli* SBS 363 was measured with the Sytox Green uptake assay. Bacterial cells were exposed to 5 μM of each ALF-derived peptide or an equal volume of sterile ultrapure water (negative control). In positive controls, peptides were substituted with 1.25 μM of magainin, a recognized pore-forming antimicrobial peptide [23].

To gain insight into the mechanism of action of the synthetic peptides, we evaluated their capacity to permeabilize the membrane of the Gram-negative *E. coli* SBS 363 by using the Sytox Green uptake assay. The increase in fluorescence was observed at the beginning of incubation with *Litvan* ALF-G_35–54_ and after less than 10 min with *Litvan* ALF-F_31–50_ (Figure 2). No significant increase in fluorescence was observed in wells incubated with sterile ultrapure water (negative control) or with *Litvan* ALF-E_33–52_, which was not active against *E. coli* SBS 363 (Figure 2; Table 1). These results indicate that these synthetic α-helical peptides are membrane-disrupting molecules with the ability to permeabilize bacterial membranes as typically observed for cAMPs.

### 3.6. Shrimp ALF-Derived Peptides Display Low Cytotoxicity to Human THP-1 Cells

The cytotoxic effect of the synthetic peptides was evaluated against the human leukemia monocytic cell line THP-1. No cytotoxic effect was observed at any of the tested concentrations (80 to 1.25 μM; cell viability > 94%) (Table 4). Due to the low influence on cell viability at the tested concentrations, it was not possible to calculate the half-maximal cytotoxic concentration (CC50) of these peptides. These results suggest that the synthetic peptides show low cytotoxicity to human cells at antibacterial and antifungal concentrations.

### 3.7. Shrimp ALF-Derived Peptides Showed No Trypanocidal or Leishmanicidal Activities

The antiparasitic effect of shrimp ALF-derived peptides was investigated against two trypanosomatid parasites: *T. cruzi* (etiologic agent of Chagas’ disease) and *L*. (*L*.) *infantum* (etiologic agent of visceral leishmaniasis). At the tested concentrations (20 to 1.25 μM), none of the synthetic peptides had any effect on either the epimastigote forms of *T. cruzi* (Tulahuen strain) or the promastigote forms of *L*. (*L*.) *infantum* (MHOM/BR/74/PP75 strain) (Table 4).

## 4. Discussion

Results showed that 20 residue linear peptides based on the central β-hairpin of the newly described *L. vannamei* ALFs from groups E to G [11] adopt an α-helix secondary structure and display a broad spectrum of activity against clinically relevant microorganisms, including multiresistant strains. Remarkably, these modified α-helical cysteine-free peptides exhibited a broader and stronger spectrum of antimicrobial activity than their correspondent forms adopting a β-hairpin structure stabilized by two cysteines [11]. Different studies have shown that the cysteine-stabilized β-hairpin is the functional domain of ALFs, which displays the ability to mimic the biological activity of the whole molecule [12,16,17]. ALFs have high binding properties for microbial components (e.g., LPS, LTA, and β-glucans) and their affinity for these components has been described as essential for their antimicrobial activities [12]. Microbial-binding involves seven charged residues located in the cysteine-stabilized β-hairpin [15], and it has been proposed that the amino acid diversity found among ALF groups in this functional domain severely impacts ALF antimicrobial activity and mechanism of action [11,12,24]. Based on the results of membrane permeabilization assays, it is most likely that the α-helical cysteine-free ALF-derived peptides have a mechanism of action similar to that of classical cationic AMPs (cAMPs) and different from whole mature ALFs. Indeed, cAMPs act on the membranes of microorganisms through electrostatic interactions with anionic phospholipids and hydrophobic interactions with the lipid bilayer, leading to their destabilization and permeabilization [25]. Thus, alterations in the primary amino acid structure represent an alternative for producing ALF-derived bioactive molecules with distinct properties and mechanisms of action.

Our antimicrobial assays revealed that *Litvan* ALF-G_35–54_ displayed the broadest and the strongest antibacterial spectrum, followed by *Litvan* ALF-F_31–50_ and then *Litvan* ALF-E_33–52_. In addition to the differences in the secondary structure, and most likely in the mechanism of action, the linear α-helical peptides synthesized in this study showed an increase in the isoelectric point compared to β-hairpin peptides [11]. As mentioned, cAMPs act on the membranes of microorganisms through electrostatic interactions and in this context the isoelectric point and hydrophobicity of the amino acid residues play an essential role in their activity [18,26,27]. The relation between the peptide sequence and activity is complex, but some generalizations can be made. For instance, increased net charge and the presence of tryptophan residues enhance antimicrobial activity through electrostatic force-associated interactions with bacterial membranes and facilitate membrane anchoring [1,28]. Therefore, the gradual increase in the isoelectric point of these peptides (from *Litvan* ALF-E_33–52_ to *Litvan* ALF-G_35–54_) and the presence of tryptophan residues in *Litvan* ALF-G_35–54_ are most likely related to the increase in antimicrobial activity observed. To date, ALFs from group B (i.e., ALF*Pm*3) display the strongest antimicrobial activity when compared to other ALF members [9,16]. Remarkably, *Litvan* ALF-G_35–54_ exhibited strong activity against Gram-positive bacteria at similar concentrations. In addition, in comparison to other crustacean AMPs displaying strong antibacterial activity (e.g., crustins, penaeidins, and armadillidins), *Litvan* ALF-G_35–54_ exhibited strong activity against both Gram-positive and Gram-negative bacteria at similar or lower concentrations [9], highlighting the strong activity of this ALF-derived peptide.

AMPs have been considered one of the most promising classes of potential drug candidates for combatting multidrug resistance [1,29,30]. AMPs show a broad spectrum of biological activities, including antiviral, antifungal, antimitogenic, anticancer, and anti-inflammatory properties. Here, we investigated the activity of linear α-helical peptides against clinically relevant bacteria and yeast, including different strains of MRSA and *Candida* spp. Remarkably, *Litvan* ALF-G_35–54_ was active against two MRSA isolates: 16003 (resistant to cefoxitin) and 17022 (resistant to cefoxitin, ciprofloxacin, clindamycin, erythromycin, gentamicin, rifampicin, and trimethoprim-sulfamethoxazole). *S. aureus* is the most frequently isolated pathogen from human skin and wound infections and the emergence of MRSA strains exhibiting resistance to conventional drugs is a significant public health challenge that requires novel therapeutic alternatives [29]. In addition, *Litvan* ALF-G_35–54_ was active against the yeasts *C. krusei* and *C. parapsilosis*, while all synthetic peptides showed activity against *C. tropicalis*. Invasive candidiasis is an important fungal disease among hospitalized patients associated with significant mortality and excessive medical costs [31]. *Candida* spp. have been reported to be significant clinical pathogens that can persist in hospital environments and are able to form biofilms on central venous catheters and other medically implanted devices [32]. Although *C. albicans* still accounts for most invasive candidiasis overall, infections caused by non-*C. albicans* species have been widely reported (reviewed in [33]). In fact, *C. parapsilosis* is often the second most commonly isolated *Candida* spp. from blood cultures and can overcome *C. albicans* in some locations [32,33]. The activity of *Litvan* ALF-G_35–54_ against these relevant clinical pathogens reinforces the potential of AMPs to replace or to be implemented together with conventional drugs to combat the issue of multidrug resistance.

In addition to their application in the clinic, recombinant or synthetic AMPs can be safely used as therapeutics in aquaculture, food additives for livestock, or food preservatives [34]. Many AMPs have been observed, when applied as food additives, to improve production performance and livestock immunity and promote intestinal health in aquaculture, as well as in poultry, swine, and ruminants breeding [34]. Our results showed that *Litvan* ALF-F_31–50_ and *Litvan* ALF-G_35–54_ could inhibit the growth of microorganisms responsible for significant loss in aquaculture, such as Gram-negative bacteria of the genus *Vibrio* [35] and the filamentous fungus *F. oxysporum* [9]. In fish farming, it has been shown that the injection of the AMPs epinecidin-1 and pleurocidin resulted in lower cumulative mortality against *Vibrio vulnificus* and *V. anguillarum* infections, respectively [36,37]. In addition, oral administration of synthetic FSB-AMP was shown to have the potential to protect *L. vannamei* shrimp against *V. parahaemolyticus* infections [38]. Altogether, these results support the applicability of shrimp ALF-derived peptides as therapeutic agents for the health of both humans and cultivated species, and future studies evaluating the effects of these peptides as therapeutics or food additives in aquaculture should be performed.

Antimicrobials are, as a class of drugs, particularly troublesome regarding cytotoxicity for hosts, since their role is to ultimately achieve microbial cell death. cAMPs have been shown to exert their antimicrobial effect by selective permeabilization of predominantly negatively charged bacterial membranes. However, their detergent-like effect can sometimes compromise both microbial and mammalian cell membranes. Our results showed that the shrimp ALF-derived peptides synthesized in this study were not active against the protozoan parasites *T. cruzi* and *L*. (*L*.) *infantum*; however, they showed low cytotoxicity to the human leukemia monocytic cell line THP-1, even at high concentrations. Peptide net charge determines the extent of the initial electrostatic interactions with both prokaryotic and eukaryotic membranes, with a more significant cationic charge favoring antimicrobial action [28]. Tailoring of these properties is likely to be the key to successfully transferring ALF-derived peptides from laboratory experiments into clinical practice as safe pharmaceutical formulations. For cAMPs, there is a correlation between a larger ratio of aromatic residues—especially tryptophan—to cationic residues and a high degree of hemolysis and cell cytotoxicity [28,39]. Hence, the absence of these residues in *Litvan* ALF-E_33–52_ and *Litvan* ALF-F_31–50_ and the low prevalence in *Litvan* ALF-G_35–54_ can contribute to the low cytotoxicity observed for these peptides.

In an era critically lacking in new antibiotics, manipulating AMPs for therapeutic application emerges as a pivotal strategy. Microorganisms’ resistance to AMPs can still remain problematic, since both Gram-positive and Gram-negative bacteria have evolved strategies to neutralize the net negative charge of their cell surfaces to avoid electrostatic interactions with cationic AMPs [40]. In addition, when activated by misfolding of outer membrane proteins, bacteria can produce factors that help to preserve and/or restore cell envelope integrity [40]. Finally, bacteria have also evolved a series of efflux pumps to transport AMPs out of their cytoplasmic space in case they have breached the bacterial membrane barriers [40]. To deal with this issue, the use of AMPs in conjunction with other antimicrobials has been considered a promising approach [3]. In this scenario, synergistic antimicrobial combinations are promising candidates that reduce potential bacterial resistance, overcome preexisting resistance to current antibiotics, prevent host toxicity, and increase antimicrobial efficacy [3]. To address this question, we evaluated the synergistic activity of the linear α-helical peptides synthesized in this study. Results showed that combinations of *Litvan* ALF-E_33–52_ or *Litvan* ALF-F_31–50_ with *Litvan* ALF-G_35–54_ acted synergistically against bacteria, while *Litvan* ALF-E_33–52_ and *Litvan* ALF-F_31–50_ acted in synergism against yeast cells. These results reveal that distinct aims can be achieved based on combinations of ALFs to improve their antimicrobial activities or to target specific microorganisms. In addition, introducing antibiotics inside bacteria has often been a challenge. We showed that ALF-derived α-helical peptides can address this challenge by disrupting bacterial membranes that might facilitate the entry of antibiotics into the cytoplasm. Therefore, combining shrimp ALF-derived peptides with other therapeutics can be an effective strategy to limit bacterial resistance through the use of different mechanisms of action.

## 5. Conclusions

We demonstrated that linear α-helical peptides inspired by the central β-hairpin of *L. vannamei* ALFs from groups E to G display a broad and interesting range of antimicrobial activities. Shrimp ALF-derived peptides were shown to be active against different bacterial and fungal strains, including human pathogens. In addition, these amphipathic AMPs were able to act synergistically to improve their antimicrobial properties and their ability to disrupt bacterial membranes. Finally, shrimp ALF-derived peptides showed low cytotoxicity to the human cell line THP-1 and were active against clinical MRSA isolates. Altogether, our results emphasize the biotechnological potential of this diverse crustacean AMP family for the development of novel therapeutic agents.

## Figures and Tables

**Table 1:** Bacteriostatic and bactericidal activities of shrimp ALF-derived peptides.

Strain	*Litvan* ALF-E_33–52_		*Litvan* ALF-F_31–50_		*Litvan* ALF-G_35–54_	
	MIC	MBC	MIC	MBC	MIC	MBC
Actinobacteria						
*Corynebacterium stationis* CIP 101282	*na*	*na*	10–20	20–40	1.25–2.5	2.5–5
*Microbacterium maritypicum* CIP 105733	20–40	20–40	5–10	5–10	1.25–2.5	1.25–2.5
*Micrococcus luteus* CIP 5345	*na*	*na*	10–20	10–20	1.25–2.5	2.5–5
Firmicutes						
*Bacillus subtilis* ATCC 6633	*na*	*na*	*na*	*na*	2.5–5	2.5–5
*Enterococcus faecalis* ATCC 29212	*na*	*na*	*na*	*na*	*na*	*na*
*Staphylococcus aureus* ATCC 25932	*na*	*na*	*na*	*na*	2.5–5	2.5–5
*Staphylococcus aureus* ATCC 29737	*na*	*na*	*na*	*na*	10–20	*na*
*Staphylococcus aureus* SG511	*na*	*na*	*na*	*na*	10–20	20–40
*Staphylococcus aureus* 16003 (MRSA)	*na*	*na*	*na*	*na*	20–40	*na*
*Staphylococcus aureus* 16006 (MRSA)	*na*	*na*	*na*	*na*	*na*	*na*
*Staphylococcus aureus* 17018 (MRSA)	*na*	*na*	*na*	*na*	*na*	*na*
*Staphylococcus aureus* 17022 (MRSA)	*na*	*na*	*na*	*na*	20–40	*na*
Proteobacteria						
*Escherichia coli* SBS 363	*na*	*na*	5–10	5–10	5–10	5–10
*Pseudomonas aeruginosa* ATCC 9027	*na*	*na*	*na*	*na*	10–20	*na*
*Vibrio alginolyticus* ATCC 17749	*na*	*na*	*na*	*na*	*na*	*na*
*Vibrio anguillarum* ATCC 19264	*na*	*na*	*na*	*na*	5–10	*na*
*Vibrio fluvialis* ENBRAPA-SE	*na*	*na*	*na*	*na*	*na*	*na*
*Vibrio harveyi* ATCC 14126	*na*	*na*	*na*	*na*	2.5–5	5–10
*Vibrio nigripulchritudo* CIP 103195	*na*	*na*	2.5–5	5–10	2.5–5	2.5–5
*Vibrio parahaemolyticus* ENBRAPA-SE	*na*	*na*	*na*	*na*	*na*	*na*
*Vibrio parahaemolyticus* IOC 18950	*na*	*na*	*na*	*na*	10–20	20–40

MIC values are reported in micromoles per liter (μM) and refer to the minimum concentration required to achieve 100% bacterial growth inhibition. MBC values (μM) refer to the minimum concentration required to kill 100% of the bacteria. MRSA, methicillin-resistant *Staphylococcus aureus*. *na*, not active at the tested concentrations.

**Table 2:** Antifungal spectra of activity of shrimp ALF-derived peptides.

Strain	*Litvan* ALF-E_33–52_		*Litvan* ALF-F_31–50_		*Litvan* ALF-G_35–54_	
	MIC	MFC	MIC	MFC	MIC	MFC
Filamentous fungi						
*Aspergillus brasiliensis* ATCC 16404	*na*	*na*	*na*	*na*	*na*	*na*
*Aspergillus niger* LAMPB-UFSC DR02	*na*	*na*	*na*	*na*	*na*	*na*
*Colletotrichum chrysophilum* MANE 147	*na*	*na*	*na*	*na*	*na*	*na*
*Colletotrichum higginsianum* MANE 166	*na*	*na*	*na*	*na*	*na*	*na*
*Fusarium oxysporum* MUCL 909	*na*	*na*	*na*	*na*	10–20	10–20
*Penicillium* sp. LIAA-UFSC	*na*	*na*	*na*	*na*	20–40	*na*
*Rhizopus* sp. LAMPB-UFSC	*na*	*na*	*na*	*na*	20–40	*na*
*Trichoderma virens* ATCC 9645	*na*	*na*	*na*	*na*	*na*	*na*
Yeast						
*Candida albicans* 12A (MDM8)	*na*	*na*	*na*	*na*	*na*	*na*
*Candida krusei* ATCC 6258	*na*	*na*	*na*	*na*	5–10	5–10
*Candida glabrata* CCT 0728	*na*	*na*	*na*	*na*	*na*	*na*
*Candida parapsilosis* ATCC 22019	*na*	*na*	*na*	*na*	20–40	*na*
*Candida tropicalis* LMC-UFSC	10–20	*na*	10–20	10–20	5–10	10–20
*Rhodotorula* sp. LIAA-UFSC	5–10	*na*	10–20	*na*	1.25–2.5	5–10
*Saccharomyces cerevisiae* CAT1	20–40	20–40	20–40	*na*	10–20	20–40

MIC values are reported in micromoles per liter (μM) and refer to the minimum concentration required to achieve 100% growth inhibition. MFC values (μM) refer to the minimum concentration required to kill 100% of the fungi. *na*, not active at the tested concentrations.

**Table 3:** Synergy of shrimp ALF-derived peptides in inhibiting microbial growth.

Peptide Combination	*M. maritypicum*	*E. coli*	*Rhodotorula* sp.
ALF-E_33–52_ + ALF-F_31–50_	2	2	0.75
ALF-E_33–52_ + ALF-G_35–54_	0.63	2	2
ALF-F_31–50_ + ALF-G_35–54_	1	0.63	2

Results are expressed as fractional inhibitory concentration (FIC) index values according to the following formula: FIC = [A]/MIC_A_ + [B]/MIC_B_, where MIC_A_ and MIC_B_ are the MICs of the synthetic peptides tested alone and [A] and [B] are the MICs of the peptides tested in combination. FIC index values are interpreted as follows: FIC ≤ 0.5, strong synergic effect; FIC = 0.5–1, synergic effect; FIC ≥ 1, additive effect; FIC = 2, no synergic effect; FIC ≥ 2, antagonist effect.

**Table 4:** Cytotoxicity and antiparasitic activity of shrimp ALF-derived peptides.

Peptide	IC50 THP-1	*T. cruzi*	*L.* (*L.*) *infantum*
*Litvan* ALF-E_33–52_	>80	*na*	*na*
*Litvan* ALF-F_31–50_	>80	*na*	*na*
*Litvan* ALF-G_35–54_	>80	*na*	*na*

Half-maximal inhibitory concentration (IC50) values are reported in micromoles per liter (μM). THP-1, human leukemia monocytic cell line; *na*, not active at the tested concentrations.

## Data Availability

Not applicable.

## Supplementary Materials

The following supporting information can be downloaded at: https://www.mdpi.com/article/10.3390/biom13010150/s1, Table S1: Strains and media.

## References

[1] Magana M., Pushpanathan M., Santos A.L., Leanse L., Fernandez M., Ioannidis A., Giulianotti M.A., Apidianakis Y., Bradfute S., Ferguson A.L. (2020). The Value of Antimicrobial Peptides in the Age of Resistance. Lancet Infect. Dis..

[2] Dijksteel G.S., Ulrich M.M.W., Middelkoop E., Boekema B.K.H.L. (2021). Review: Lessons Learned From Clinical Trials Using Antimicrobial Peptides (AMPs). Front. Microbiol..

[3] Duong L., Gross S.P., Siryaporn A. (2021). Developing Antimicrobial Synergy With AMPs. Front. Med. Technol..

[4] Lazzaro B.P., Zasloff M., Rolff J. (2020). Antimicrobial Peptides: Application Informed by Evolution. Science.

[5] Björn C., Noppa L., Näslund Salomonsson E., Johansson A.-L., Nilsson E., Mahlapuu M., Håkansson J. (2015). Efficacy and Safety Profile of the Novel Antimicrobial Peptide PXL150 in a Mouse Model of Infected Burn Wounds. Int. J. Antimicrob. Agents.

[6] Hou M., Zhang N., Yang J., Meng X., Yang R., Li J., Sun T. (2013). Antimicrobial Peptide LL-37 and IDR-1 Ameliorate MRSA Pneumonia in Vivo. Cell. Physiol. Biochem..

[7] Segev-Zarko L., Saar-Dover R., Brumfeld V., Mangoni M.L., Shai Y. (2015). Mechanisms of Biofilm Inhibition and Degradation by Antimicrobial Peptides. Biochem. J..

[8] Greber K.E., Dawgul M. (2017). Antimicrobial Peptides Under Clinical Trials. Curr. Top. Med. Chem..

[9] Matos G.M., Rosa R.D. (2022). On the Silver Jubilee of Crustacean Antimicrobial Peptides. Rev. Aquac..

[10] Destoumieux-Garzón D., Rosa R.D., Schmitt P., Barreto C., Vidal-Dupiol J., Mitta G., Gueguen Y., Bachère E. (2016). Antimicrobial Peptides in Marine Invertebrate Health and Disease. Philos. Trans. R. Soc. B Biol. Sci..

[11] Matos G.M., Schmitt P., Barreto C., Farias N.D., Toledo-Silva G., Guzmán F., Destoumieux-Garzón D., Perazzolo L.M., Rosa R.D. (2018). Massive Gene Expansion and Sequence Diversification Is Associated with Diverse Tissue Distribution, Regulation and Antimicrobial Properties of Anti-Lipopolysaccharide Factors in Shrimp. Mar. Drugs.

[12] Rosa R.D., Vergnes A., de Lorgeril J., Goncalves P., Perazzolo L.M., Sauné L., Romestand B., Fievet J., Gueguen Y., Bachère E. (2013). Functional Divergence in Shrimp Anti-Lipopolysaccharide Factors (ALFs): From Recognition of Cell Wall Components to Antimicrobial Activity. PLoS ONE.

[13] Ponprateep S., Tharntada S., Somboonwiwat K., Tassanakajon A. (2012). Gene Silencing Reveals a Crucial Role for Anti-Lipopolysaccharide Factors from Penaeus Monodon in the Protection against Microbial Infections. Fish Shellfish. Immunol..

[14] Yang Y., Boze H., Chemardin P., Padilla A., Moulin G., Tassanakajon A., Pugnière M., Roquet F., Destoumieux-Garzón D., Gueguen Y. (2009). NMR Structure of RALF-Pm3, an Anti-Lipopolysaccharide Factor from Shrimp: Model of the Possible Lipid A-Binding Site. Biopolymers.

[15] Schmitt P., Rosa R.D., Destoumieux-Garzón D. (2016). An Intimate Link between Antimicrobial Peptide Sequence Diversity and Binding to Essential Components of Bacterial Membranes. Biochim. Biophys. Acta BBA—Biomembr..

[16] Somboonwiwat K., Marcos M., Tassanakajon A., Klinbunga S., Aumelas A., Romestand B., Gueguen Y., Boze H., Moulin G., Bachère E. (2005). Recombinant Expression and Anti-Microbial Activity of Anti-Lipopolysaccharide Factor (ALF) from the Black Tiger Shrimp Penaeus Monodon. Dev. Comp. Immunol..

[17] Yang H., Li S., Li F., Xiang J. (2016). Structure and Bioactivity of a Modified Peptide Derived from the LPS-Binding Domain of an Anti-Lipopolysaccharide Factor (ALF) of Shrimp. Mar. Drugs.

[18] Guo S., Li S., Li F., Zhang X., Xiang J. (2014). Modification of a Synthetic LPS-Binding Domain of Anti-Lipopolysaccharide Factor from Shrimp Reveals Strong Structure-Activity Relationship in Their Antimicrobial Characteristics. Dev. Comp. Immunol..

[19] Tharntada S., Somboonwiwat K., Rimphanitchayakit V., Tassanakajon A. (2008). Anti-Lipopolysaccharide Factors from the Black Tiger Shrimp, Penaeus Monodon, Are Encoded by Two Genomic Loci. Fish Shellfish. Immunol..

[20] de la Vega E., O’Leary N.A., Shockey J.E., Robalino J., Payne C., Browdy C.L., Warr G.W., Gross P.S. (2008). Anti-Lipopolysaccharide Factor in Litopenaeus Vannamei (LvALF): A Broad Spectrum Antimicrobial Peptide Essential for Shrimp Immunity against Bacterial and Fungal Infection. Mol. Immunol..

[21] Hetru C., Bulet P. (1997). Strategies for the Isolation and Characterization of Antimicrobial Peptides of Invertebrates. Methods Mol. Biol..

[22] Schmitt P., de Lorgeril J., Gueguen Y., Destoumieux-Garzón D., Bachère E. (2012). Expression, Tissue Localization and Synergy of Antimicrobial Peptides and Proteins in the Immune Response of the Oyster Crassostrea Gigas. Dev. Comp. Immunol..

[23] Löfgren S.E., Miletti L.C., Steindel M., Bachère E., Barracco M.A. (2008). Trypanocidal and Leishmanicidal Activities of Different Antimicrobial Peptides (AMPs) Isolated from Aquatic Animals. Exp. Parasitol..

[24] Li S., Guo S., Li F., Xiang J. (2015). Functional Diversity of Anti-Lipopolysaccharide Factor Isoforms in Shrimp and Their Characters Related to Antiviral Activity. Mar. Drugs.

[25] Brogden K.A. (2005). Antimicrobial Peptides: Pore Formers or Metabolic Inhibitors in Bacteria?. Nat. Rev. Microbiol..

[26] Jiang Z., Vasil A.I., Hale J.D., Hancock R.E.W., Vasil M.L., Hodges R.S. (2008). Effects of Net Charge and the Number of Positively Charged Residues on the Biological Activity of Amphipathic Alpha-Helical Cationic Antimicrobial Peptides. Biopolymers.

[27] Leptihn S., Har J.Y., Wohland T., Ding J.L. (2010). Correlation of Charge, Hydrophobicity, and Structure with Antimicrobial Activity of S1 and MIRIAM Peptides. Biochemistry.

[28] Feng X., Jin S., Wang M., Pang Q., Liu C., Liu R., Wang Y., Yang H., Liu F., Liu Y. (2020). The Critical Role of Tryptophan in the Antimicrobial Activity and Cell Toxicity of the Duck Antimicrobial Peptide DCATH. Front. Microbiol..

[29] Mohamed M.F., Abdelkhalek A., Seleem M.N. (2016). Evaluation of Short Synthetic Antimicrobial Peptides for Treatment of Drug-Resistant and Intracellular Staphylococcus Aureus. Sci. Rep..

[30] Divyashree M., Mani M.K., Reddy D., Kumavath R., Ghosh P., Azevedo V., Barh D. (2020). Clinical Applications of Antimicrobial Peptides (AMPs): Where Do We Stand Now?. Protein Pept. Lett..

[31] Kullberg B.J., Arendrup M.C. (2015). Invasive Candidiasis. N. Engl. J. Med..

[32] Zoppo M., Poma N., Di Luca M., Bottai D., Tavanti A. (2021). Genetic Manipulation as a Tool to Unravel Candida Parapsilosis Species Complex Virulence and Drug Resistance: State of the Art. J. Fungi.

[33] Trofa D., Gácser A., Nosanchuk J.D. (2008). Candida Parapsilosis, an Emerging Fungal Pathogen. Clin. Microbiol. Rev..

[34] Huan Y., Kong Q., Mou H., Yi H. (2020). Antimicrobial Peptides: Classification, Design, Application and Research Progress in Multiple Fields. Front. Microbiol..

[35] Morales-Covarrubias M.S., Cuéllar-Anjel J., Varela-Mejías A., Elizondo-Ovares C. (2018). Shrimp Bacterial Infections in Latin America: A Review. Asian Fish. Sci..

[36] Pan C.-Y., Chen J.-Y., Cheng Y.-S.E., Chen C.-Y., Ni I.-H., Sheen J.-F., Pan Y.-L., Kuo C.-M. (2007). Gene Expression and Localization of the Epinecidin-1 Antimicrobial Peptide in the Grouper (Epinephelus Coioides), and Its Role in Protecting Fish against Pathogenic Infection. DNA Cell Biol..

[37] Jia X., Patrzykat A., Devlin R.H., Ackerman P.A., Iwama G.K., Hancock R.E.W. (2000). Antimicrobial Peptides Protect Coho Salmon FromVibrio Anguillarum Infections. Appl. Environ. Microbiol..

[38] Cheng A.-C., Lin H.-L., Shiu Y.-L., Tyan Y.-C., Liu C.-H. (2017). Isolation and Characterization of Antimicrobial Peptides Derived from Bacillus Subtilis E20-Fermented Soybean Meal and Its Use for Preventing Vibrio Infection in Shrimp Aquaculture. Fish Shellfish. Immunol..

[39] Strøm M.B., Rekdal O., Svendsen J.S. (2002). Antimicrobial Activity of Short Arginine- and Tryptophan-Rich Peptides. J. Pept. Sci..

[40] Destoumieux-Garzón D., Duperthuy M., Vanhove A.S., Schmitt P., Wai S.N. (2014). Resistance to Antimicrobial Peptides in Vibrios. Antibiotics.

