# Supplementary material for: Antimicrobial Spectrum of Activity and Mechanism of Action of Linear Alpha-Helical Peptides Inspired by Shrimp Anti-Lipopolysaccharide Factors
Source: Biomolecules. 2023 Jan 11;13(1):150. doi: 10.3390/biom13010150 (PMC9856130; doi:10.3390/biom13010150)
Supplement: Supplementary file 1 [file biomolecules-13-00150-s001.zip › biomolecules-2144350-supplementary/biomolecules-2144350-Table S1 - conversion.pdf]

Table S1. Strains and media

Strain	Source	Origin	Culture medium for growth	Culture medium for antimicrobial assays	Temperature	Comments
**Gram-positive bacteria**						
Bacillus subtilis ATCC 6633	Ref	ATCC	LB	PB	37 °C	Used in sterility testing
Corynebacterium stationis CIP 101282	Ref	Pasteur Institute	Zobell	Zobell 1/3	30 °C	Isolated from film of marine fouling organisms
Enterococcus faecalis ATCC 29212	Ref	ATCC	LB	PB	37 °C	Isolated from urine and has applications in food testing, media testing, quality control, and water testing
Microbacterium maritypicum CIP 105733	Ref	Pasteur Institute	Zobell	Zobell 1/3	30 °C	Isolated from seawater and marine mud
Micrococcus luteus CIP 5345	Ref	Pasteur Institute	LB	PB	30 °C	Renamed *Kocuria rhizophila*
Staphylococcus aureus SG511	Ref	UM	LB	PB	37 °C	Used as a standard strain for antimicrobial susceptibility testing
Staphylococcus aureus ATCC 25932	Ref	ATCC	LB	PB	37 °C	Clinical isolate used as a quality control strain for some tests
Staphylococcus aureus ATCC 29737	Ref	ATCC	LB	PB	37 °C	Strain FDA used in sterility testing and susceptibility disc testing
Staphylococcus aureus 16003	MRSA	UFSC	LB	PB	37 °C	Isolated from a tracheal secretion of a hospitalized patient (resistant to cefoxitin)
Staphylococcus aureus 16006	MRSA	UFSC	LB	PB	37 °C	Isolated from the blood of a hospitalized patient (resistant to cefoxitin, ciprofloxacin, clindamycin and erythromycin)

Strain	Source	Origin	Culture medium for growth	Culture medium for antimicrobial assays	Temperature	Comment
Staphylococcus aureus 17018	MRSA	UFSC	LB	PB	37 °C	Isolated from a tracheal aspirate of a hospitalized patient (resistant to cefoxitin, ciprofloxacin, clindamycin, erythromycin and gentamicin)
Staphylococcus aureus 17022	MRSA	UFSC	LB	PB	37 °C	Isolated from a bone fragment of a hospitalized patient (resistant to cefoxitin, ciprofloxacin, clindamycin, erythromycin, gentamicin, rifampicin and trimethoprim-sulfamethoxazole)
**Gram-negative bacteria**						
Escherichia coli SBS 363	Ref	Pasteur Institute	LB	PB	37 °C	Used as a standard strain for antimicrobial susceptibility testing
Pseudomonas aeruginosa ATCC 9027	Ref	ATCC	LB	PB	37 °C	Isolated from an outer ear infection used in efficacy testing, media testing, and preparatory test control
Vibrio alginolyticus ATCC 17749	Ref	ATCC	LB-NaCl	PB-NaCl	20 °C	Isolated in Japan from spoiled horse mackerel that resulted in food poisoning
Vibrio anguillarum ATCC 19264	Ref	ATCC	LB-NaCl	PB-NaCl	20 °C	Type strain isolated from ulcerous lesion in cod
Vibrio fluvialis EMBRAPA-SE	Env	EMBRAPA	LB-NaCl	PB-NaCl	20 °C	Environmental strain isolated from diseased shrimp

Strain	Source	Origin	Culture medium for growth	Culture medium for antimicrobial assays	Temperature	Comment
Vibrio harveyi ATCC 14126	Ref	ATCC	LB-NaCl	PB-NaCl	20 °C	Type strain isolated from a dead luminescing amphipod, *Talorchestia* sp.
Vibrio nigripulchritudo CIP 103195	Ref	Pasteur Institute	LB-NaCl	PB-NaCl	28 °C	Isolated from seawater enriched with chitin
Vibrio parahaemolyticus IOC 18950	Ref	IOC	LB-NaCl	PB-NaCl	20 °C	A standard strain from an outbreak in Cascavel (Brazil)
Vibrio parahaemolyticus EMBRAPA-SE	Env	EMBRAPA	LB-NaCl	PB-NaCl	20 °C	Environmental strain isolated from diseased shrimp
**Filamentous fungi**						
Aspergillus brasiliensis ATCC 16404	Ref	ATCC	PDA	½ PDB	28 °C	Isolated from blueberry
Aspergillus niger LAMPB-UFSC DR02	Env	LAMPB	PDA	½ PDB	28 °C	Endophytic fungi of *Platanus orientalis*
Colletotrichum chrysophilum MANE 147	Ref	MANE	PDA	½ PDB	28 °C	Isolated from apple leaf/Gala
Colletotrichum higginsianum MANE 166	Ref	MANE	PDA	½ PDB	28 °C	Isolated from *Arabidopsis thaliana*
Fusarium oxysporum MUCL 909	Ref	UM	PDA	½ PDB	28 °C	Shrimp opportunistic pathogen
Penicillium sp. LIAA-UFSC	Env	LIAA	PDA	½ PDB	28 °C	Environmental strain isolated from shrimp midgut
Rhizopus sp. LAMPB-UFSC	Env	LAMPB	PDA	½ PDB	28 °C	Environmental strain isolated from air
Trichoderma virens ATCC 9645	Ref	ATCC	PDA	½ PDB	28 °C	Isolated from soil
**Yeast**						
Candida albicans 12A (MDM8)	Ref	Butantan Institute	Sabouraud	Sabouraud	28 °C	Standard strain used in antimicrobial tests

Strain	Source	Origin	Culture medium for growth	Culture medium for antimicrobial assays	Temperature	Comment
Candida krusei ATCC 6258	Ref	ATCC	Sabouraud	Sabouraud	28 °C	Quality control strain isolated from sputum of patient with bronchomycosis
Candida glabrata CCT 0728	Ref	CCT	Sabouraud	Sabouraud	28 °C	-
Candida parapsilosis ATCC 22019	Ref	ATCC	Sabouraud	Sabouraud	28 °C	Type strain for industrial biotechnology and quality control isolated from case of sprue
Candida tropicalis LMC-UFSC	Ref	UFSC	Sabouraud	Sabouraud	28 °C	-
Rhodotorula sp. LIAA-UFSC	Env	LIAA	Sabouraud	Sabouraud	28 °C	Environmental strain isolated from shrimp midgut
Saccharomyces cerevisiae CAT1	Ref	UFSC	Sabouraud	Sabouraud	28 °C	Industrial fuel-ethanol fermentative strain

ATCC, American Type Culture Collection; CCT, Collection of Tropical Cultures; CIP, Collection de l'Institut Pasteur; EMBRAPA, Empresa Brasileira de Pesquisa Agropecuária; Env, environmental isolate; IOC, Oswaldo Cruz Institute; LAMPB, Laboratory of Microorganisms and Biotechnological Processes; LIAA, Laboratory of Immunology Applied to Aquculture; MANE, Micoteca Anne Löre Schroeder; MRSA: Methicillin-resistant *Staphylococcus aureus*; MUCL, Université Catholique de Louvain; Ref, reference strain; UFSC, Federal University of Santa Catarina; UM, University of Montpellier. Comment on and origins have been indicated where available.

Luria-Bertani (LB) medium (10 g/L bactopeptone, 5 g/L yeast extract, 10 g/L NaCl, pH 7.2). Poor broth (PB) medium (10 g/L bactopeptone, 10 g/L NaCl, pH 7.2). LB-NaCl (10 g/L bactopeptone, 5 g/L yeast extract, 30 g/L NaCl, pH 7.2). PB-NaCl (10 g/L bactopeptone, 30 g/L NaCl, pH 7.2). Zobell medium (4 g/L bactopeptone, 1 g/L yeast extract, 400 mM NaCl, 20 mM KCl, 5 mM $MgSO_4$, 1.4 mM $CaCl_2$, pH 7.2). Zobell 1/3 medium (1.3 g/L bactopeptone, 0.33 g/L yeast extract, 400 mM NaCl, 20 mM KCl, 5 mM $MgSO_4$, 1.4 mM $CaCl_2$, pH 7.2). Sabouraud medium (10 g/L bactopeptone, 40 g/L dextrose, pH 5.6). Potato Dextrose Agar (PDA, 26 g/L Potato Dextrose Broth, 1.5% agar, pH 5.6). ½ Potato Dextrose Broth (½ PDB, 13.5 g/L Potato Dextrose Broth, pH 5.6).
